# Supplementary material for: Long-range order enhance performance of patterned blue quantum dot light-emitting diodes
Source: Nat Commun. 2025 Aug 16;16:7643. doi: 10.1038/s41467-025-62345-1 (PMC12357929; doi:10.1038/s41467-025-62345-1)
Supplement: Supplementary file 1 — Supplementary Information [file 41467_2025_62345_MOESM1_ESM.pdf]

Supplementary Information

Long-Range Order Enhance Performance of Patterned Blue Quantum Dot Light-Emitting Diodes

Yuyu Jia[1,2], Hui Li[3]*, Ning Guo[2,4], Fengmian Li[2,5], Tianchen Li[2,4], Haoran Ma[1], Yuyan Zhao[3], Hanfei Gao[3], Dan Wang[1], Jiangang Feng[3], Zhiyuan He[1]*, Lei Jiang[2,3] and Yuchen Wu[2,3,4]*

[1]School of Materials Science and Engineering, Beijing Institute of Technology, Beijing 100081, P. R. China

[2]Key Laboratory of Bio-inspired Materials and Interfacial Science, Technical Institute of Physics and Chemistry, Chinese Academy of Sciences, Beijing 100190, P. R. China

[3]Suzhou Institute for Advanced Research, University of Science and Technology of China, Suzhou, Jiangsu 215123, P. R. China

[4]University of Chinese Academy of Sciences (UCAS) Beijing 100049, P. R. China

[5]Key Laboratory of Bio-Inspired Smart Interfacial Science and Technology of Ministry of Education, School of Chemistry, Beihang University, Beijing 100191, P. R. China

*Corresponding author. Emails: lihui17703806212@163.com (H.L.); hezy@bit.edu.cn (Z.H.); wuyuchen@iccas.ac.cn (Y.W.)

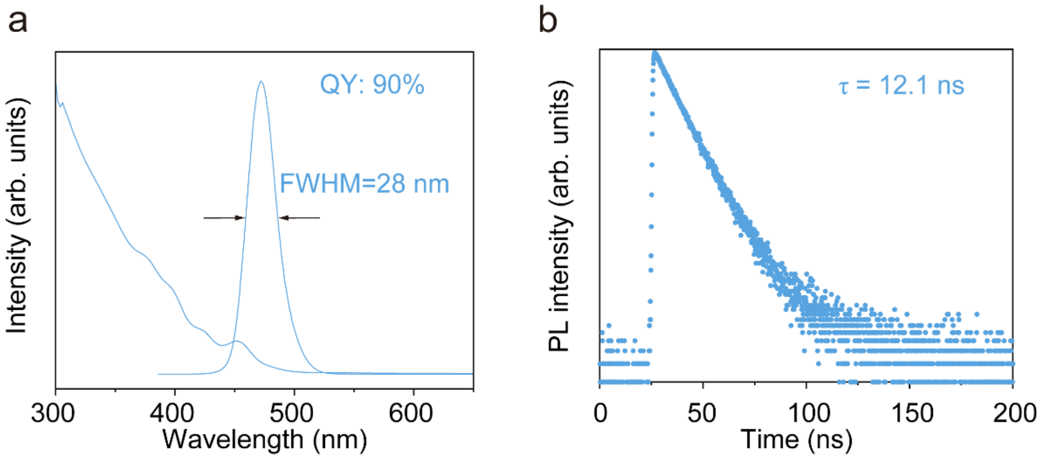

Supplementary Fig. 1 The optical performance of OA modified QD solutions. a, The fluorescence spectrum, absorption spectrum and PL QY of the OA modified QD solution. **b,** The transient PL decay of the OA modified QD solution.

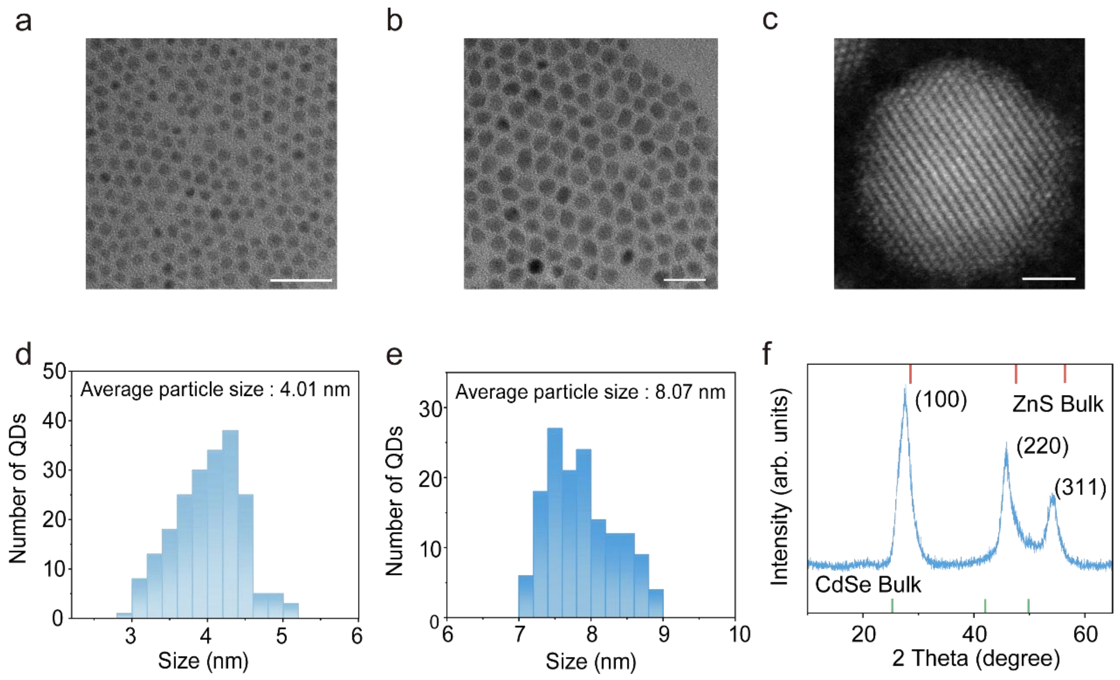

Supplementary Fig. 2 TEM and XRD of OA modified QDs. a, TEM image of the core of QDs (scale bar is 20 nm). **b,** TEM images of OA modified QDs (scale bar is 20 nm). **c,** Atomically resolved HAADF-STEM image of the OA modified QDs (scale bar is 2 nm). **d,** Particle size distribution the core of QDs. **e,** Particle size distribution of OA modified QDs. **f,** XRD of OA modified QDs.

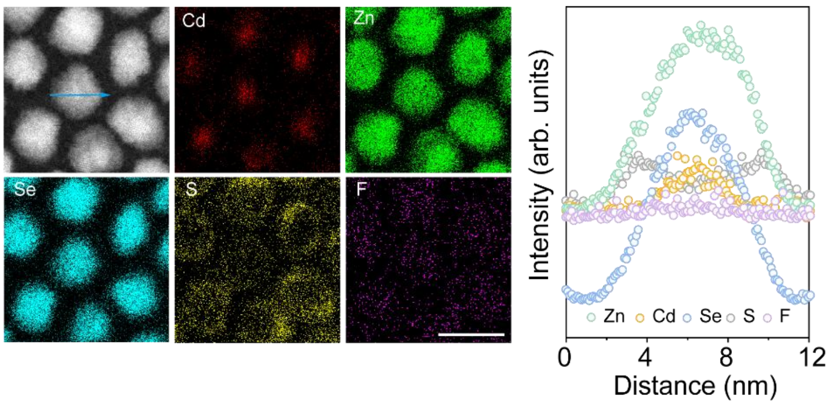

Supplementary Fig. 3 Elemental distribution of 3-F-CA modified QDs. STEM image of 3-F-CA modified QDs and elemental mapping of Cd, Zn, Se, S and F in the same area using energy dispersive X-ray spectroscopy (EDS, left image, scale bar is 10 nm), with line profiles showing the distribution of elements in 3-F-CA modified QDs (right image).

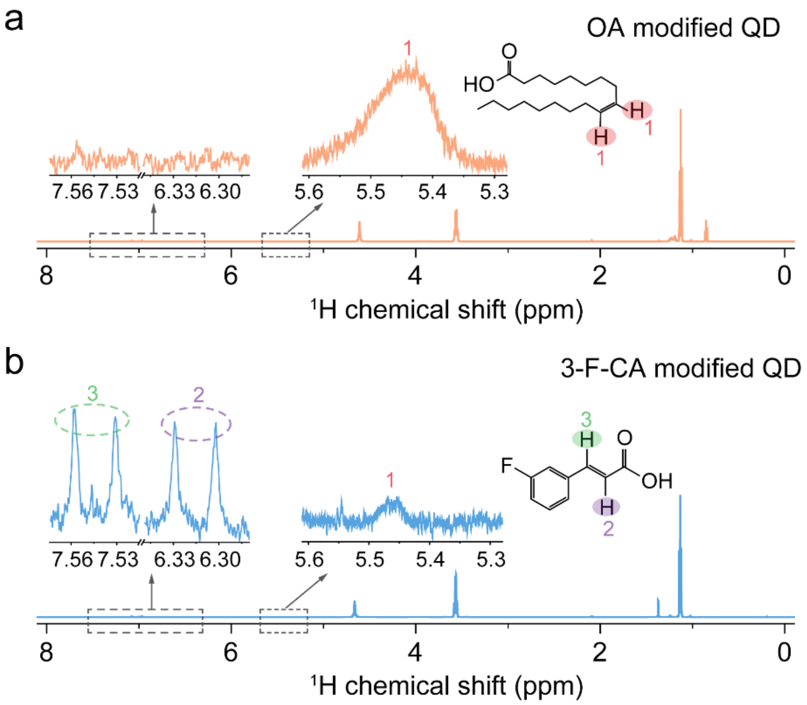

Supplementary Fig. 4 NMR spectra. ^{1}H NMR spectra of **a,** OA and **b,** 3-F-CA modified QDs. After 3-F-CA ligand exchange, purification was performed using ethanol and deuterated toluene.

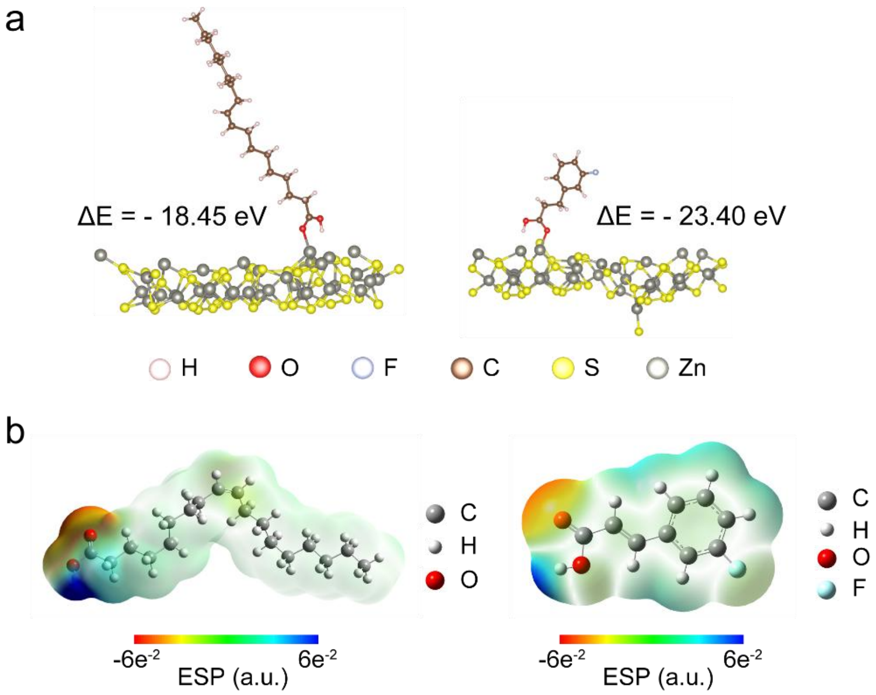

Supplementary Fig. 5 Binding energy and electrostatic potential. a, Adsorption model of OA molecule and 3-F-CA molecule on the QD surface and their corresponding binding energies. **b,** ESP maps of OA and 3-F-CA.

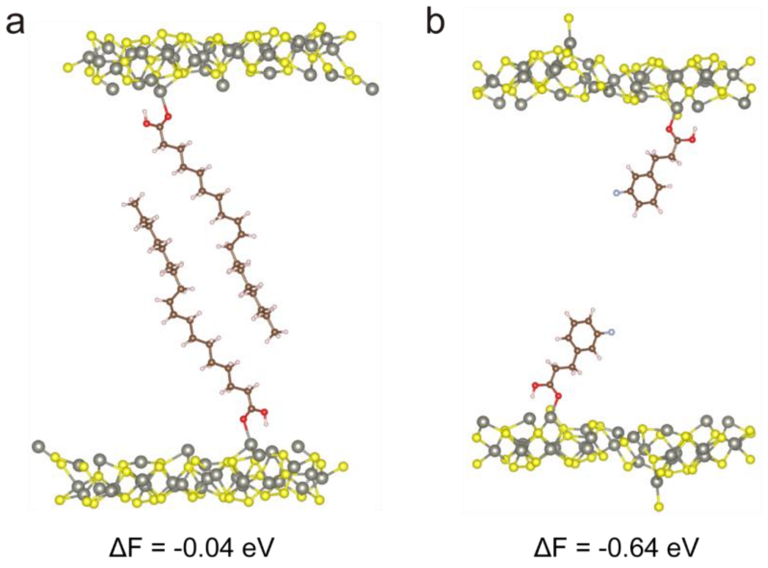

Supplementary Fig. 6 The interactions between QDs. a, The interaction energy between an OA molecule modified QD surface and a nearby QD surface. **b,** The interaction energy between a 3-F-CA molecule modified QD surface and a nearby QD surface.

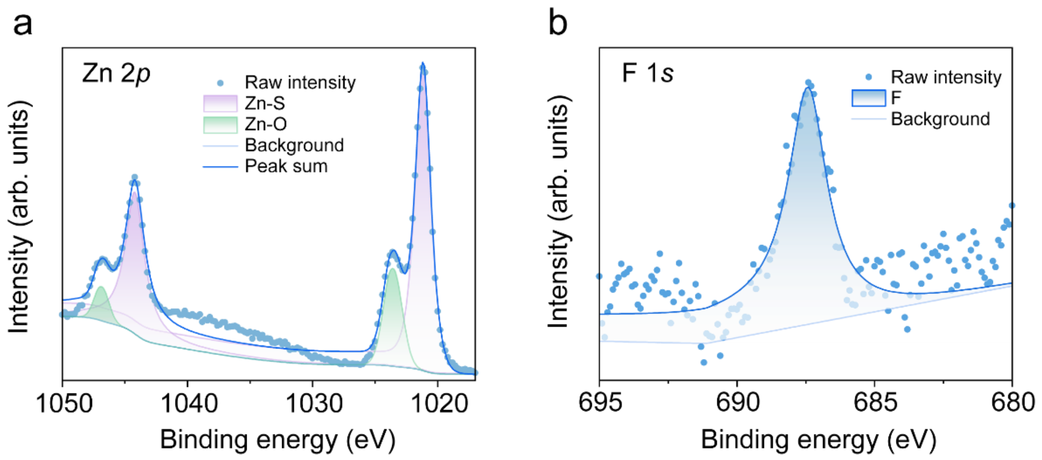

Supplementary Fig. 7 XPS spectra a, XPS spectra of Zn 2*p* orbit of the 3-F-CA modified QD film. b, XPS spectra of F 1*s* orbit of the 3-F-CA modified QD film. The Zn 2*p* orbital signals originated from the outermost ZnS layer of the QDs (purple peaks) and Zn bound to carboxylic acid (-COOH) groups (green peaks). The integrated peak area of F was utilized to quantify the relative amount of 3-F-CA ligands. The Zn and F peaks were integrated, and the corresponding atomic ratio was calculated by normalizing with their XPS sensitivity factors. The atomic ratio was determined using the following formula:

$$R_{\mathrm{X:Y}}=\frac{\frac{A_{\mathrm{X}}}{S_{\mathrm{X}}}}{\frac{A_{\mathrm{Y}}}{S_{\mathrm{Y}}}}$$

where A_{X} and A_{Y} are the XPS peak areas of elements X and Y obtained from spectral integration, and S_{X} and S_{Y} are the sensitivity factors for elements X and Y.

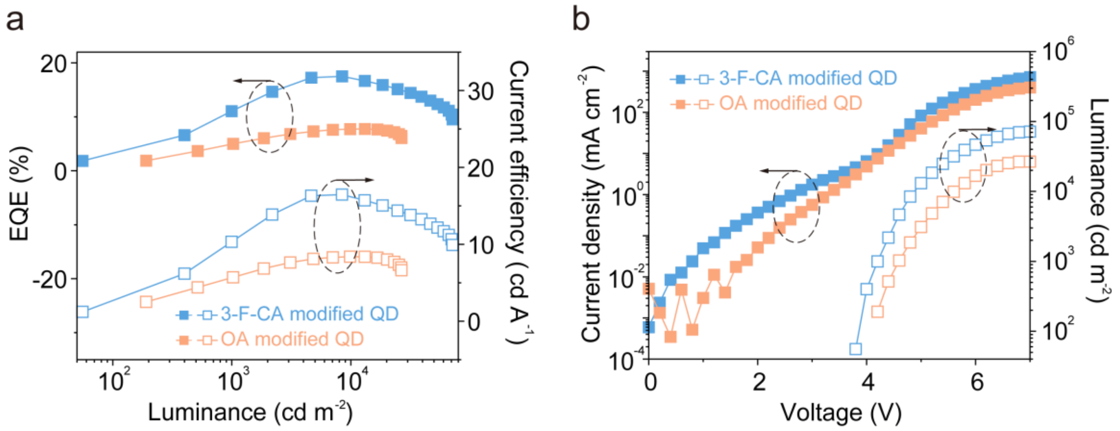

Supplementary Fig. 8 The performance of QLEDs fabricated by the spin-coating. **a,** EQE-current efficiency-luminance characteristics and **b,** *J*-*L*-*V* characteristics of QLEDs based on 3-F-CA modified QD film and OA modified QD film prepared by spin-coating.

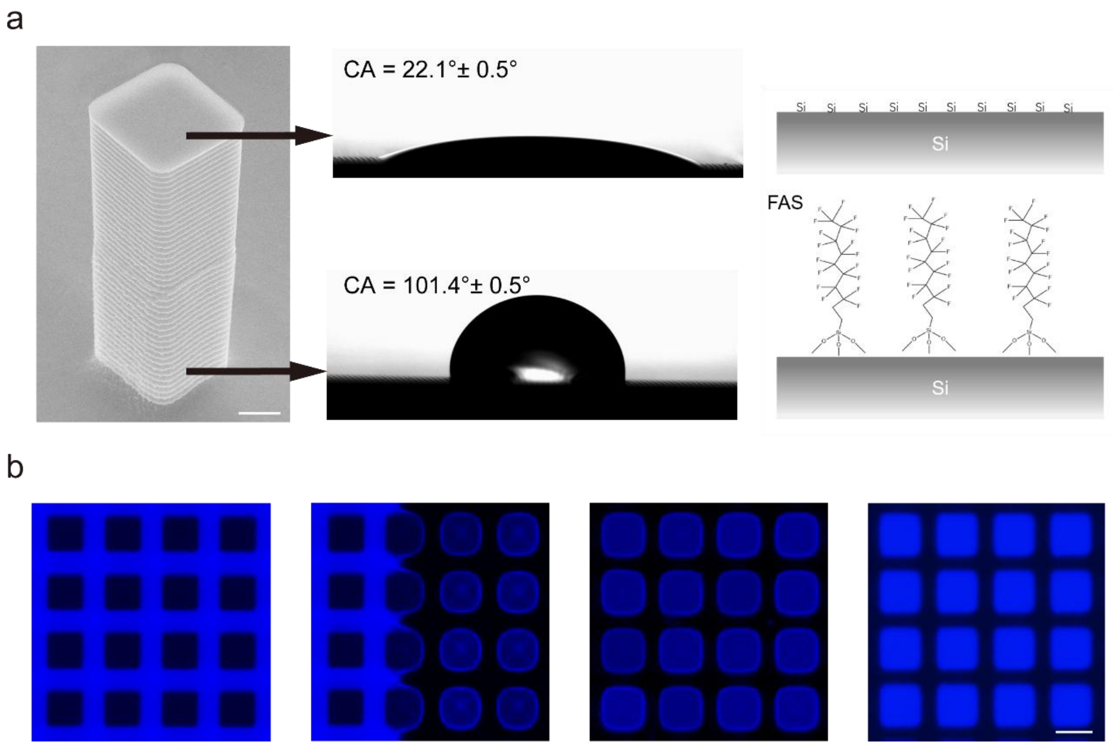

Supplementary Fig. 9 Modification of micropillar and the dewetting process of QDs. a, The contact angle (CA) of the micropillar's top is 22.1° ± 0.5°, exhibiting the hydrophilicity. The side of the micropillar shows hydrophobicity with the CA of 101.4° ± 0.5° (scale bar is 2 μm). **b,** Assembly processes of QD microstructure arrays under in-situ fluorescence microscopy (scale bar is 5 μm).

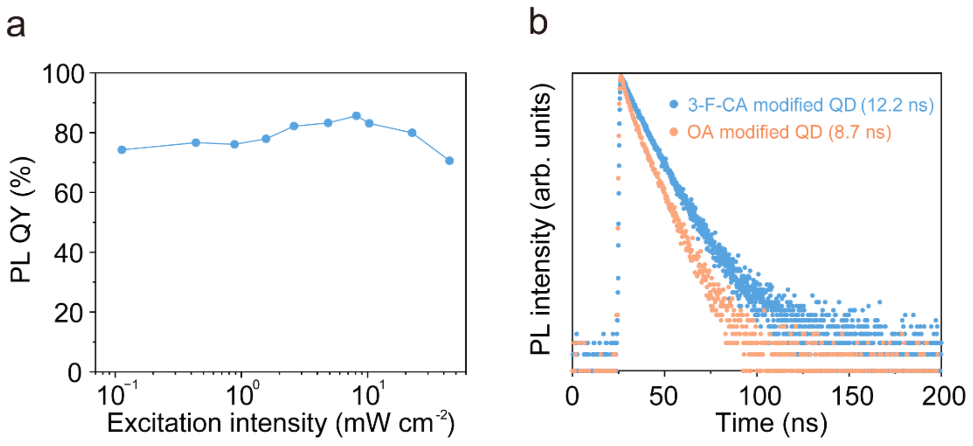

Supplementary Fig. 10 Optical properties of the QD layer self-assembled. a, PL QY as a function of excitation intensity for the 3-F-CA modified QD layer. **b,** The transient PL decay of the OA modified QD layer and the 3-F-CA modified QD layer. All of them are assembled by capillary bridge.

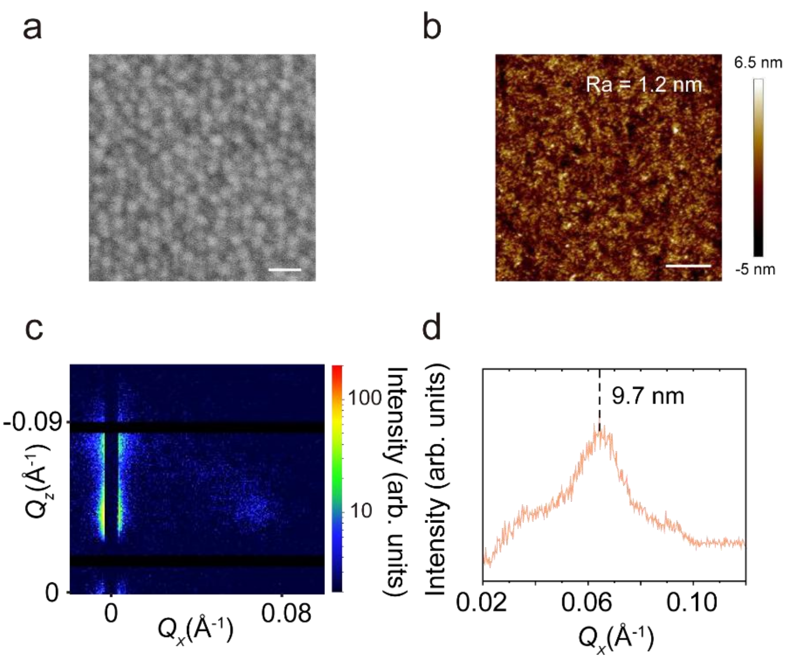

Supplementary Fig. 11 The performance of the self-assembled OA modified QD layer. a, SEM image of the OA modified QD layer (scale bar is 20 nm). **b,** AFM image of the OA modified QD layer (scale bar is 1 μm). **c,** GISAXS pattern of the OA modified QD layer. **d,** Azimuthal integration of GISAXS diffraction patterns from OA modified QD layer. All of them are assembled by capillary bridge confinement.

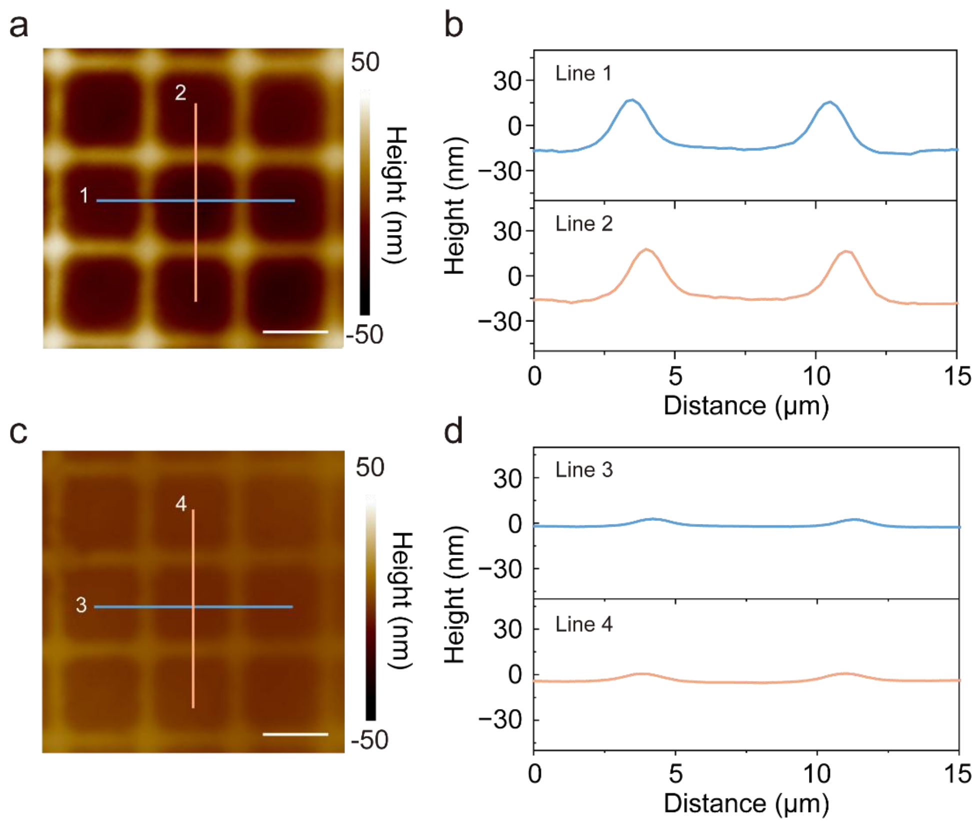

Supplementary Fig. 12 Surface roughness of pixel. a, AFM image of photoresist layer before the deposition of QDs (scale bar is 5 μm) and **b,** corresponding hight profile along the blue and orange solid line. **c,** AFM image of photoresist layer after the deposition of QDs (scale bar is 5 μm) and **d,** corresponding hight profile along the blue and orange line.

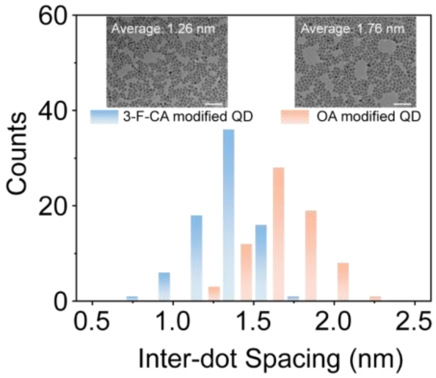

Supplementary Fig. 13 The interparticle spacing of QDs. The distance between OA modified QDs and 3-F-CA modified QDs. Inset, TEM images of OA modified QDs and 3-F-CA modified QDs (scale bar is 50 nm).

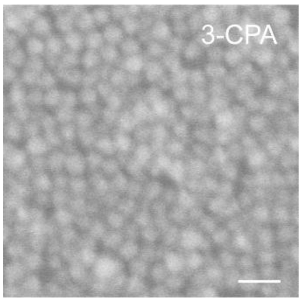

Supplementary Fig. 14 SEM image. SEM image of 3-CPA modified QDs. (scale bar is 20 nm).

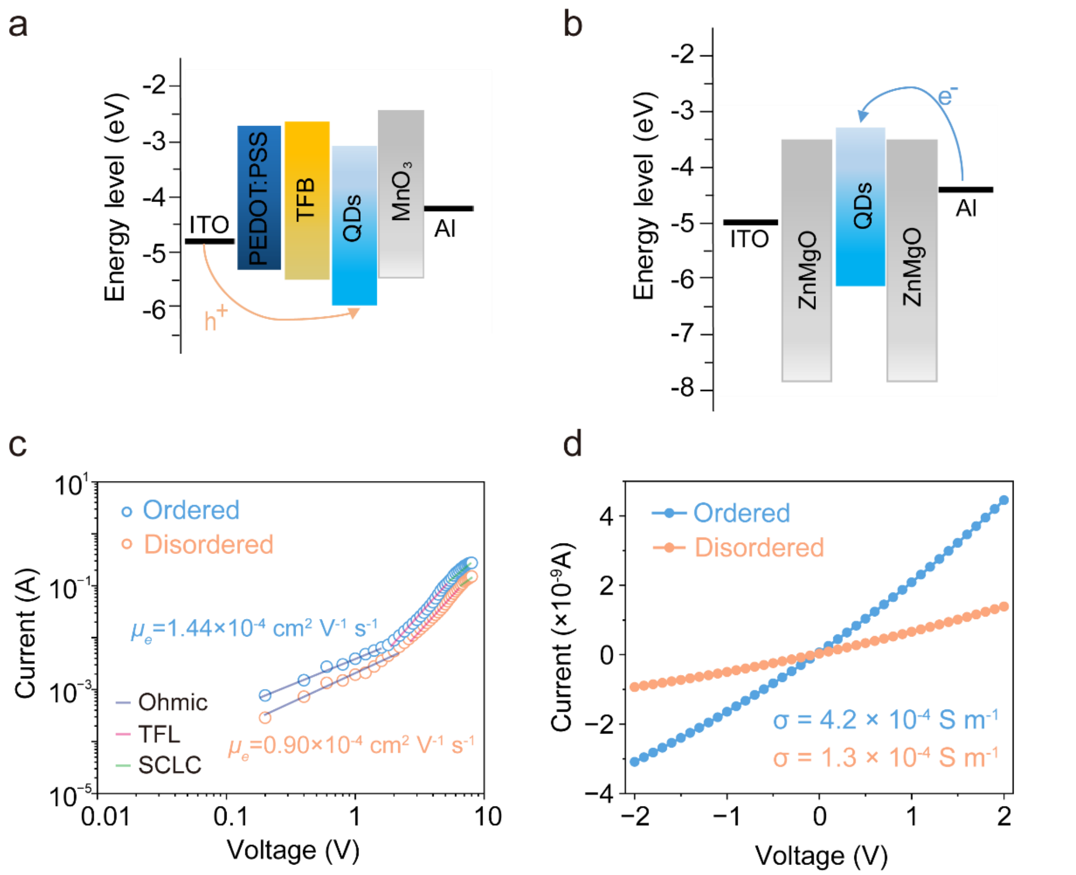

Supplementary Fig. 15 Electrical properties of ordered and disordered QD layers. **a,** Energy level diagram of each functional layer in hole-only device. **b,** Energy level diagram of each functional layer in electron-only device. **c,** Space charge-limited current measurements of electron-only devices based on OA modified disordered QDs and 3-F-CA modified ordered QDs. **d,** Field-effect transistors of ordered and disordered QD films.

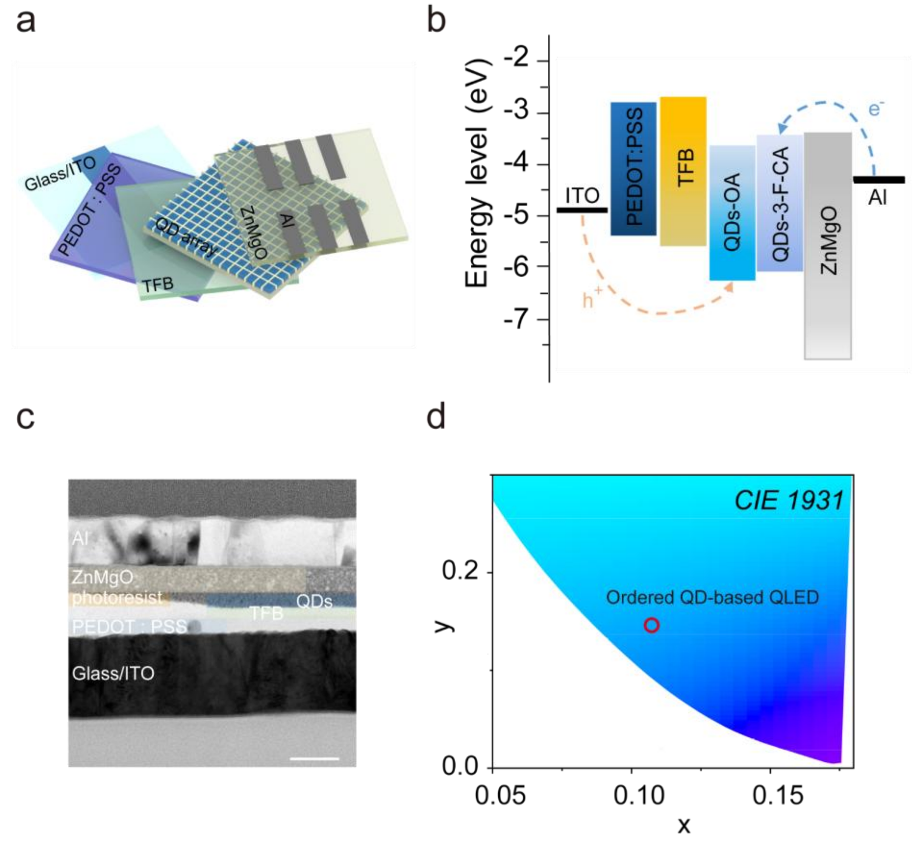

Supplementary Fig. 16 Device with QD microstructure arrays. a, A schematic diagram of the QLED structure is shown, with the QD layer prepared from QD microstructure arrays. **b,** Energy level diagram of the QDs and different functional layers of the device. **c,** TEM image of QLED cross-section (scale bar is 100 nm). **d,** The *CIE*-1931 coordinates of ordered QD-based QLED.

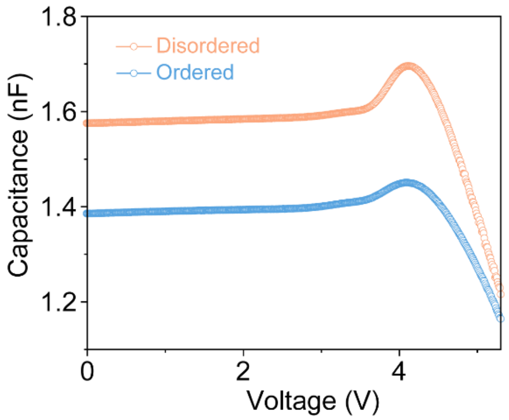

Supplementary Fig. 17 *C-V* curve of QLEDs. The capacitance-voltage characteristics of ordered and disordered QD-based QLEDs.

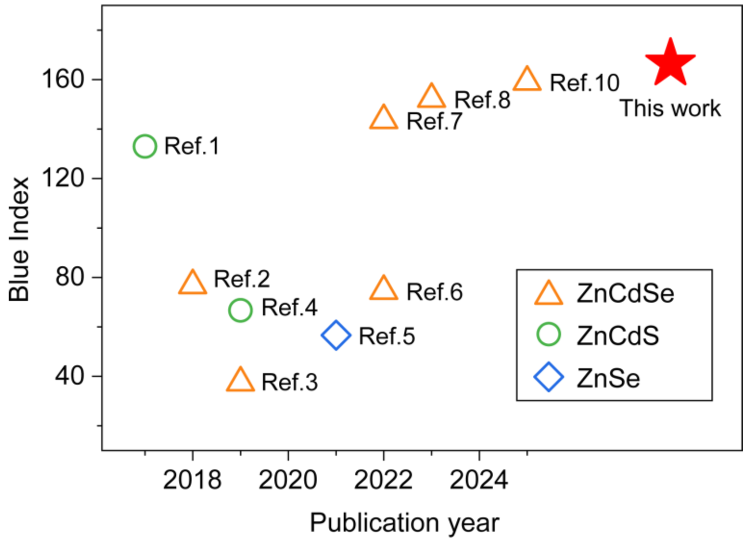

Supplementary Fig. 18 Research progress on the blue index of blue Cd-based QLEDs. The asterisk denotes the work presented in this paper. Blue index is defined as the ratio of current efficiency and *CIE*-y.

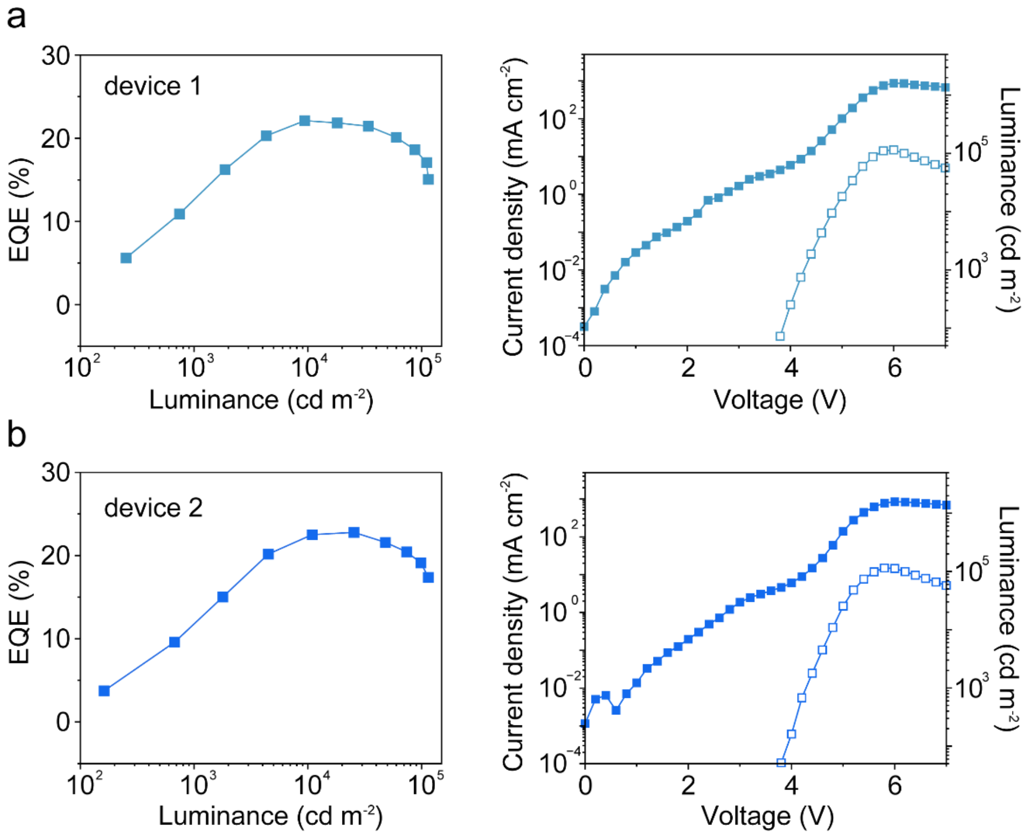

Supplementary Fig. 19 The electrical performance of QLEDs. EQE-luminance characteristics curves and current density-luminance-voltage characteristics curves of **a,** device 1 and **b,** device 2.

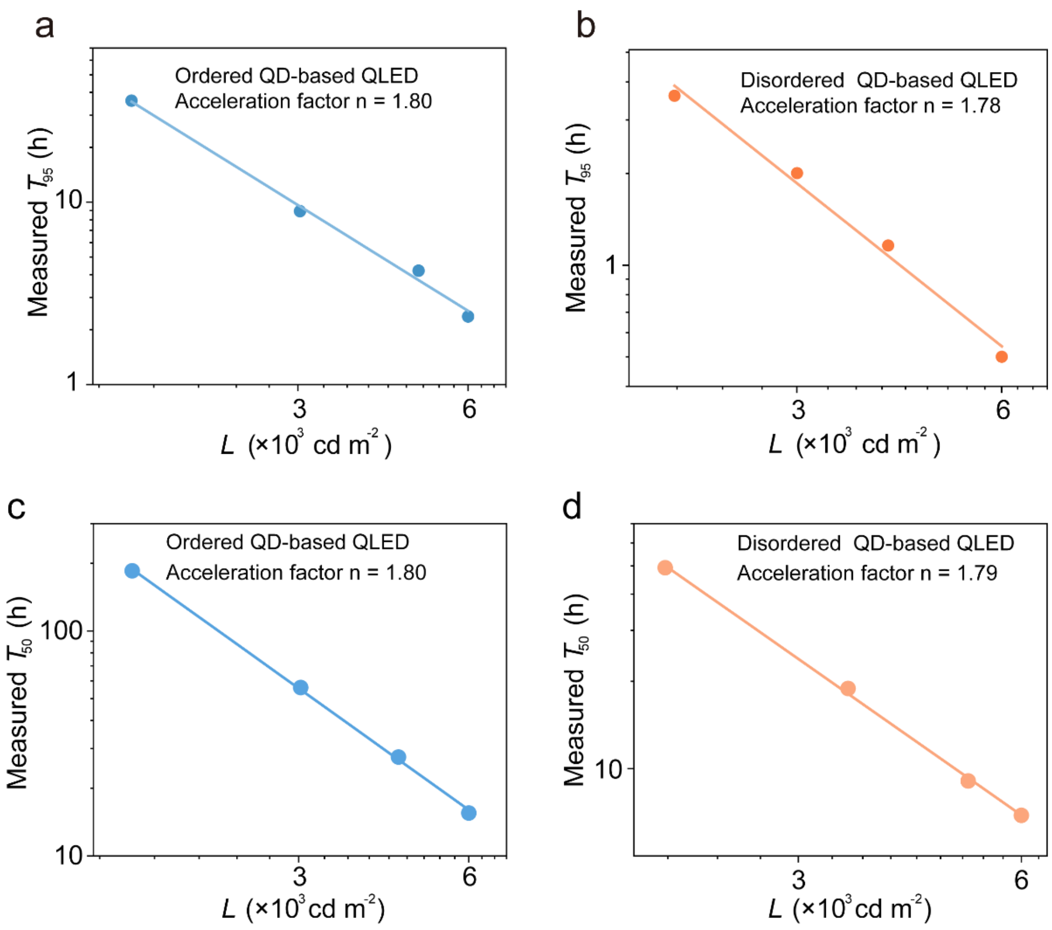

Supplementary Fig. 20 Extrapolation of accelerating factor (n) for the lifetime estimation. Extrapolation of accelerating factor (n) for the lifetime estimation by fitting the Log(T_{95})-Log(L_0) data points of **a,** ordered QD-based QLED, and **b,** disordered QD-based QLED. Extrapolation of accelerating factor (n) for the lifetime estimation by fitting the Log(T_{50})-Log(L_0) data points of **c,** ordered QD-based QLED, and **d,** disordered QD-based QLED.

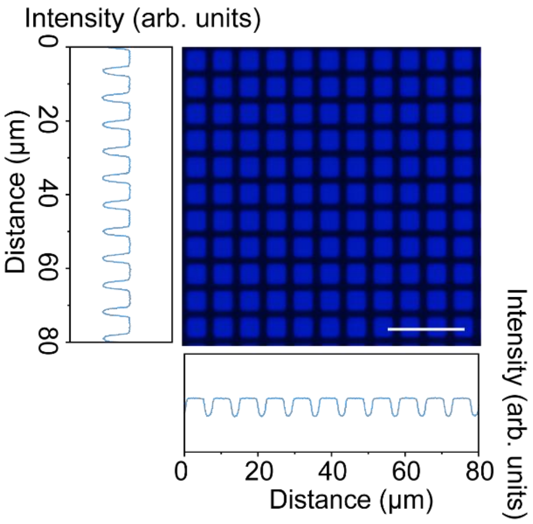

Supplementary Fig. 21 The EL intensity of QLED. Ordered QD-based QLED under confocal microscopy showing EL intensity (scale bar is 20 µm).

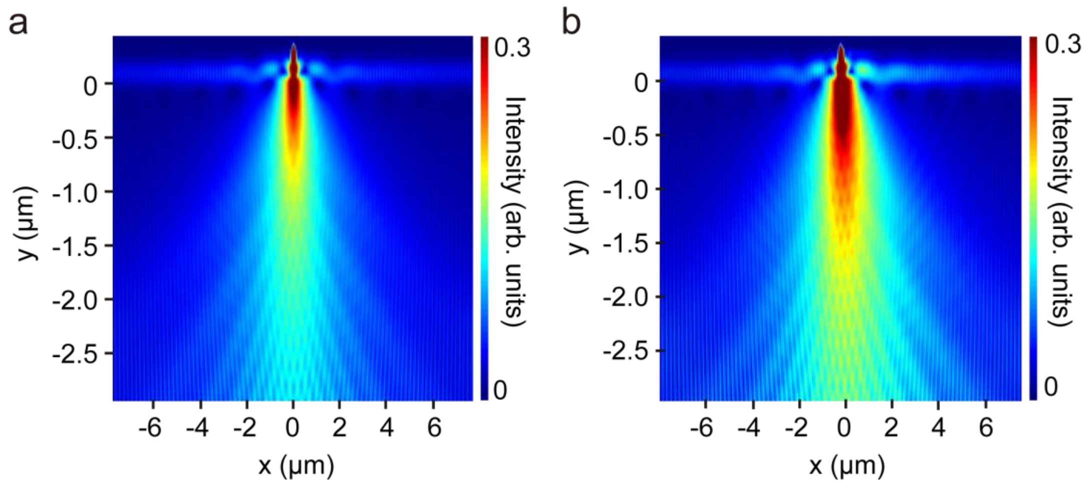

Supplementary Fig. 22 The intensity field distribution of QLEDs. Intensity field distribution of the QLED **a,** without and **b,** with microstructure.

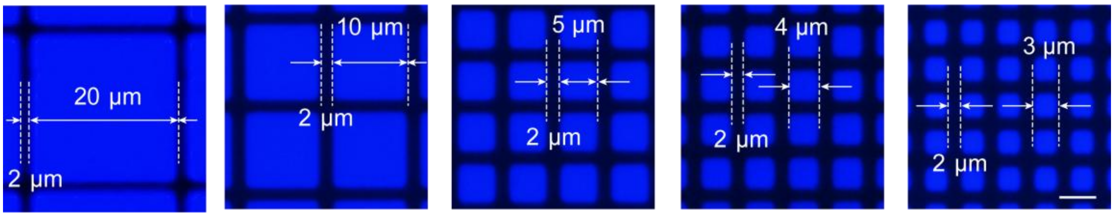

Supplementary Fig. 23 High-resolution EL microscopic images of a QLEDs. EL microscopic images of QLEDs with different pixel size (20 μm×20 μm, 10 μm×10 μm, 5 μm×5 μm, 4 μm×4 μm, 3 μm×3 μm, scale bar is 5 μm).

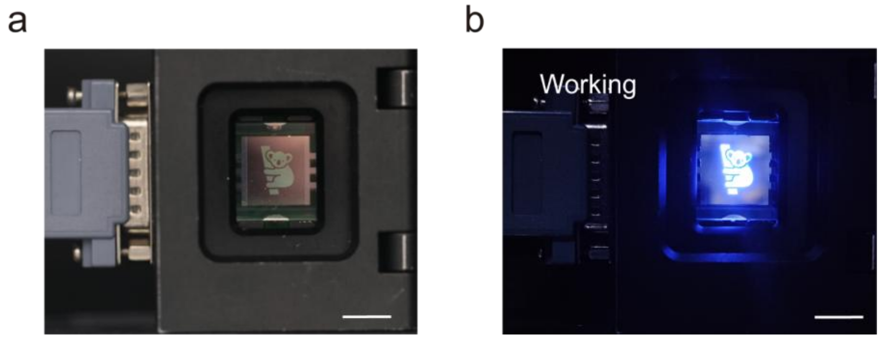

Supplementary Fig. 24 Patterned QLED. a, Photograph of the high-resolution QLED (scale bar is 1 cm). **b,** Working state of the high-resolution QLED (scale bar is 1 cm).

Supplementary Table 1. Atomic ratios of surface elements in the 3-F-CA modified QD film derived from XPS results.

Element	Area	RSF	Atomic ratio
Zn (Zn-O)	46,669	28.72	1
F	6,044	4.43	0.84

Supplementary Table 2. Performance comparison of the devices developed in this work with other high- performance blue QLEDs.

**Ref.**	**EL (nm)**	**EQE (%)**	L_{Max} **(cd m^{-2})**	$\eta_{A\ Max}$ **(cd A^{-2})**	***CIE-y***	**Blue Index**	T_{95} **(h)**
**1**	445	11.9	10,400	2.26	0.017	133	4
**2**	479	16.2	14,100	11.83	0.154	77	-
**3**	476	8.0	62,600	6.93	0.185	37	0.2
**4**	452	17.4	7,992	2	0.03	66	-
**5**	445	12.2	1,000	1.7	0.03	56	-
**6**	470	21.9	11,000	19.9	0.267	74	57
**7**	475	20.6	2,920	19.1	0.133	143	4
**8**	471	20.8	10,000	12.33	0.081	152	227
**9**	458	23	20,000	-	0.04	-	87
**10**	472	23.5	36810	17.5	0.11	159	-
**This work**	476	24.1	101,519	23.3	0.14	166	54

Supplementary Note 1

The calculation of carrier mobility:

The *I*-*V* behavior of the devices exhibits three regions, each defined by a slope *k*: the ohmic region (k = 1), the trap-filled limit (TFL) region (k > 3), and the space-charge-limited current (SCLC) region with no traps, which increases with applied bias (k = 2). Here, the trap density N_t is linearly proportional to trap-filled limit voltage V_{TFL} at which a transition of *I*-*V* behavior from ohmic to TFL occurs:

$$V_{\mathrm{TFL}} = N_{\mathrm{t}}\frac{eL^2}{2\varepsilon\varepsilon_0} \tag{1}$$

where e is electron charge, L is the thickness of the QD layer, ε is the relative dielectric constant, ε_0 is the vacuum permittivity. The mobility μ for each device by fitting the single trap level model:

$$J = \frac{9\varepsilon\varepsilon_0\mu\theta V^2}{8L^3} \tag{2}$$

where J is the current density, V is the applied voltage, and θ is the trapping factor given by

$$\theta = (\frac{N_{\mathrm{v}}}{N_{\mathrm{t}}})\exp(\frac{-E_{\mathrm{t}}}{k_{\mathrm{B}}T}) \tag{3}$$

where N_v is the density of states, k_B is Boltzmann constant, E_t is the trap at energy obtained by fitting the *I*-*V* curves at different temperatures using the Arrhenius equation.

Supplementary Note 2

The calculation of conductivity:

We fabricated field-effect transistors (FET) to determine the conductivity of the QD films. The channel length (L) and width (W) of the transistors were 2 μm and 40 μm, respectively. The conductivity (σ) of the two QD films was calculated using the following equation:

$$\sigma = \frac{I}{V} \times \frac{L}{T \times W} \tag{4}$$

where L is the channel length, W is the channel width, T is the thickness of the QD film, and I/V is the slope of the current-voltage curve.

Supplementary Note 3

The calculation of the active area of QLED under operating conditions:

In patterned QLED devices, the regions covered by insulating photoresist layers are electrically non-conductive, necessitating the exclusion of these areas during device performance characterization. Consequently, the active area (S_l) is calculated using the following formula:

$$S_l = S \times \frac{S_\mathrm{a}}{S_\mathrm{b}} \quad (5)$$

where S denotes the total device area (i.e., the overlapping region between cathode and anode), which was fixed at 3.0 mm^2 in our experimental configuration; S_a represents the area of individual pixel; S_b denotes the area of individual repeating unit.

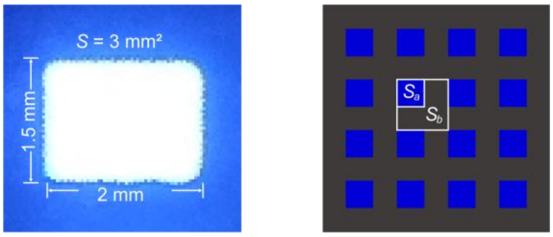

Supplementary Fig. 25 The active area of QLED. Image of QLED under operating conditions (left) and schematic diagram of its active area calculation (right).

Supplementary Note 4

The calculation of resolution:

Resolution is conventionally quantified in pixels per inch (PPI), representing the number of individual pixels contained within one inch:

$$\mathrm{PPI} = \frac{\sqrt{X^2+Y^2}}{Z} \tag{6}$$

where X is the number of horizontal pixels, Y is the number of vertical pixels, and Z is the diagonal size of the screen (inch).

Supplementary References

1. Shen H, *et al.* Efficient and long-lifetime full-color light-emitting diodes using high luminescence quantum yield thick-shell quantum dots. *Nanoscale* **9**, 13583-13591 (2017).
2. Lin Q, *et al.* Nonblinking Quantum-Dot-Based Blue Light-Emitting Diodes with High Efficiency and a Balanced Charge-Injection Process. *ACS Photon.* **5**, 939-946 (2018).
3. Shen H, *et al.* Visible quantum dot light-emitting diodes with simultaneous high brightness and efficiency. *Nat. Photon.* **13**, 192-197 (2019).
4. Li D, *et al.* Blue quantum dot light-emitting diodes with high luminance by improving the charge transfer balance. *Chem. Commun.* **55**, 3501-3504 (2019).
5. Gao M, *et al.* Bulk-like ZnSe Quantum Dots Enabling Efficient Ultranarrow Blue Light-Emitting Diodes. *Nano Lett.* **21**, 7252-7260 (2021).
6. Deng Y, *et al.* Solution-processed green and blue quantum-dot light-emitting diodes with eliminated charge leakage. *Nat. Photon.* **16**, 505-511 (2022).
7. Wang F, *et al.* High-Performance Blue Quantum-Dot Light-Emitting Diodes by Alleviating Electron Trapping. *Adv. Opt. Mater.* **10**, 2200319 (2022).
8. Chen X, *et al.* Blue light-emitting diodes based on colloidal quantum dots with reduced surface-bulk coupling. *Nat. Commun.* **14**, 284 (2023).
9. Zhang W, *et al.* Stable and efficient pure blue quantum-dot LEDs enabled by inserting an anti-oxidation layer. *Nat. Commun.* **15**, 783 (2024).
10. Ren Y, *et al.* In Situ, Treatment with Guanidinium Chloride Ligand Enables Efficient Blue Quantum Dot Light-Emitting Diodes with 23.5% External Quantum Efficiency. *Adv. Mater.* **37**, 2413183 (2025).
